# A cost effective RFLP method to genotype *Solute carrier organic anion 1B1* (*SLCO1B1)* c.1929A>C (p.Leu643Phe, rs34671512); a variant with potential effect on rosuvastatin pharmacokinetics

**DOI:** 10.1186/s13104-018-3469-4

**Published:** 2018-06-14

**Authors:** Nyarai D. Soko, Collen Masimirembwa, Collet Dandara

**Affiliations:** 1 Division of Human Genetics, Department of Pathology, Faculty of Health Sciences, Anzio Road, Observatory, Cape Town, South Africa; 2 African Institute of Biomedical Science and Technology (AiBST), Wilkins Hospital, Corner J. Tongogara and Princes Road, Harare, Zimbabwe

**Keywords:** *SLCO1B1*, Rosuvastatin, African, rs34671512, RFLP, Pharmacogenetics, Pharmacokinetics, SLCO1B1*22, SLCO1B1*35

## Abstract

**Objective:**

This study describes a restriction fragment polymorphism protocol for rapidly screening the polymorphism *SLCO1B1 c.1929A*>*C* in genomic DNA samples. The polymorphism *SLCO1B1 c.1929A*>*C* has been associated with increased activity resulting in increased hepatic uptake of drugs. Currently *SLCO1B1 c.1929A*>*C* is genotyped using direct sequencing techniques and 5′ nuclease based assays which can be cost prohibiting in resource limited settings. The aim of this study therefore was to design and validate a cost effective RFLP for genotyping the *SLCO1B1 c.1929A*>*C* polymorphism. This study was designed to investigate the effect of the polymorphism *SLCO1B1 c.1929A*>*C* on interindividual variability in rosuvastatin pharmacokinetics in healthy volunteers of African descent.

**Results:**

We describe a restriction fragment length polymorphism method to genotype *SLCO1B1 c.1929A*>*C* polymorphism using the restriction enzyme *Ase1*. A student’s *t* test with Welch correction was used to establish association between the *SLCO1B1 c.1929A*>*C* variant and rosuvastatin exposure. The frequency of the *SLCO1B1 c.1929C* allele amongst Zimbabweans was 6%. The *SLCO1B1 c.1929C* allele was associated with a 75% reduction (P < 0.001) in rosuvastatin exposure when compared to individuals carrying the wild type *SLCO1B1 c.1929A* allele. Polymorphism *c.1929A*>*C* may therefore play a significant role in rosuvastatin response. The RFLP method is quick and cost effective.

## Introduction

As part of a larger pharmacogenetics study, we opted to explore the possible impact of single nucleotide polymorphism c.1929A>C; within the gene *Solute carrier organic anion transporter family member 1B1* (*SLCO1B1*) on rosuvastatin pharmacokinetics. The *SLCO1B1* (*c.1929A*>*C, p.Leu643Phe*) SNP has been associated with altered OATP1B1 transporter activity [1]. The *c.1929C* allele has been associated with increased hepatic uptake of the antifolate methotrexate (P = 0.028) in cancer patients [2] and increased uptake of atorvastatin [3]. To do this we required a quick and cheap method to detect *SLCO1B1 c.1929A*>*C* in our study population. Currently, genotyping of the *SLCO1B1 c.1929A*>*C* is done using the relatively more expensive methods such as Sanger sequencing, 5′-nuclease assays [3], microarrays [2, 3]. Although methods such as mass arrays and sequencing offer more robust, automated and at times pre-validated options, restriction fragment length polymorphism (RFLP) remains an attractive method of choice for resource limited settings [4]. We describe, therefore, a quick cost effective RFLP based method to detect the *SLCO1B1* c.1929A>C variant.

## Main text

### Methods

#### Design of the RFLP

Both forward and reverse PCR primers were designed using the PrimerQuest Tool (Integrated DNA Technology, Iowa, USA) and tested for specificity using the NCBI Nucleotide Blast tool. The forward primer was 5′-GGCCAGAGGCAACTAGAGTAT-3′ and 5′-ATACTCTAGTTGCCTCTGGCC -3′ served as the reverse primer. Using the web based program NebCutter [5]; we selected the restriction enzyme *Ase1* for the production of restriction fragments.

#### Evaluation of the RFLP

To evaluate the RFLP we selected 157 genomic DNA samples from an in-house DNA bank wholly owned by the Pharmacogenomics and Cancer Research Group, in the Human Genetics Division, Faculty of Health Sciences at the University of Cape Town. All DNA samples have approval for use in ongoing pharmacogenomics research. All 157 DNA samples were obtained from Zimbabwean adults of self-reported African-Bantu descent. Ethnicity could be traced to the third generation.

Amongst these 157 individuals were 30 who took part in a pharmacokinetic study of rosuvastatin. Recruitment of healthy volunteers for the rosuvastatin study has been reported elsewhere [6], briefly 30 healthy adult male (age 18–30 years) volunteers were recruited from the Harare Metropolitan Province, in Zimbabwe. Ethical approval for the pharmacokinetic trial was obtained from the Medicines Research Council of Zimbabwe (Ref: MRCZ/A/1793), The University of Cape Town Faculty of Health Sciences Research Ethics Committee (Ref: 197/2014) and the Chitungwiza General Hospital Institution Review Board. After a 12 h overnight fast, each participant administered 20 mg of rosuvastatin calcium with 450 ml of water. Venous blood was collected over 30 h post drug administration and drug levels from each time point were estimated and the resultant drug concentrations used to develop pharmacokinetic profiles of each individual. Rosuvastatin plasma concentrations were analysed using ultra performance liquid chromatography (UPLC) with mass spectrometric detection according to a method developed and validated by Astra Zeneca (Sweden) with a limit of detection of quantification for rosuvastatin in plasma was 0.47 ng/ml.

Genomic DNA was extracted from 1.5 ml of whole blood drawn from each participant using the QIAmp DNA Blood Kit (Qiagen, Hilden, Germany). The PCR proceeded as follows: each 25 µl reaction contained 75 ng of genomic DNA, 0.2× GoTaq PCR Buffer (Promega, Wisconsin, USA), 0.2 mM dNTPs (ThermoFisher Scientific, Massachusetts, USA), 1.0 µM of each primer (Integrated DNA Technology, Iowa, USA), 0.5 U of GoTaq (Promega, Wisconsin, USA), made up to 25 µl using nuclease free water. The reaction began with an initial denaturation at 94 °C for 5 min, followed by 40 cycles of denaturation at 94 °C for 45 s, annealing at 61.0 °C for 30 s, extension at 72 °C for 1 min and a final extension at 72 °C for 5 min. The PCR product was 470 bp in length.

**Fig. 1:**
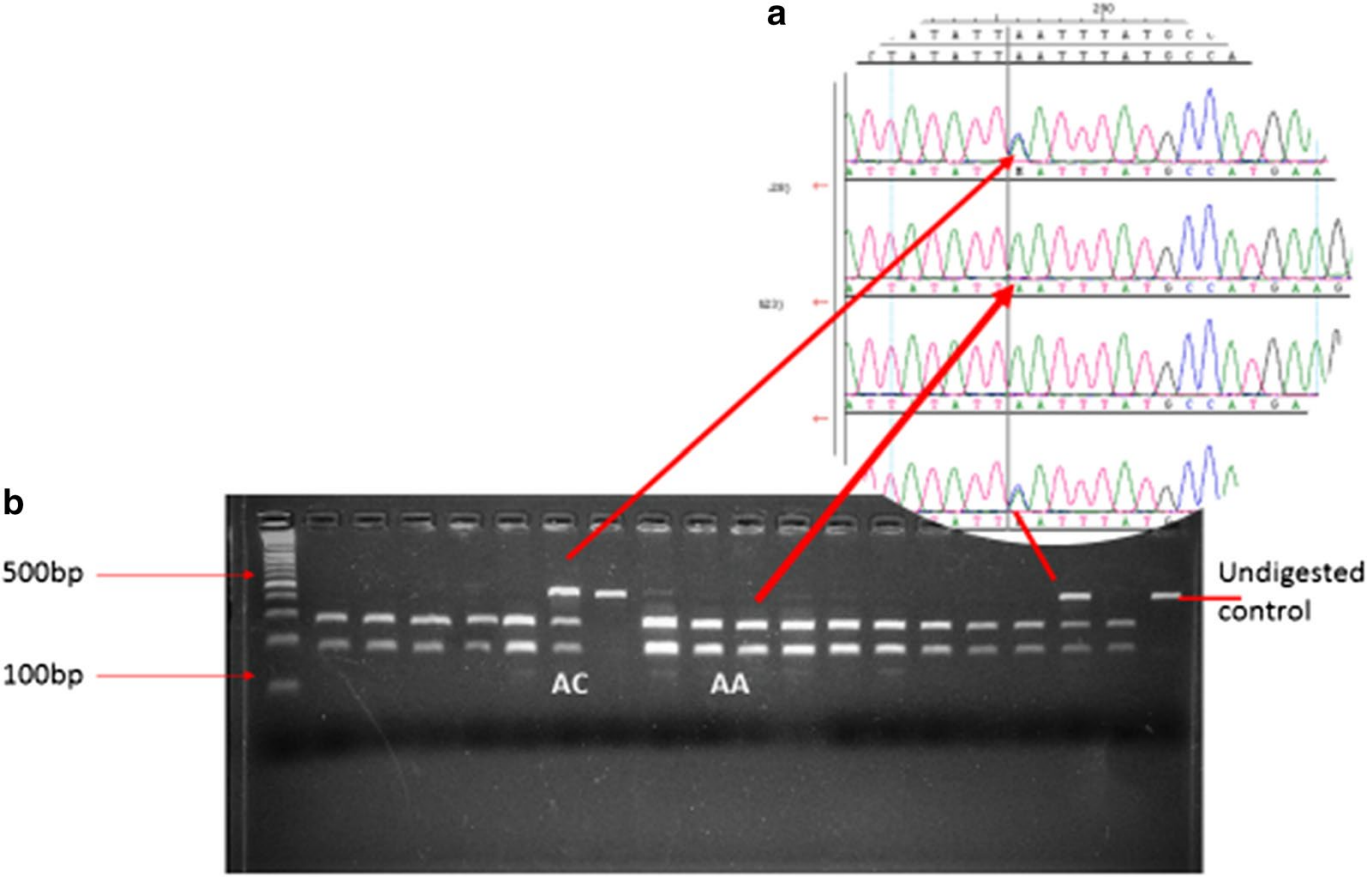
**a** Genotype validation by Sanger sequencing and **b** RFLP digest showing the AC genotype (digest yields 470, 282 and 188 bp fragments), the AA genotype (digest yields 282 and 188 bp) and undigested 470 bp controls

The 470 bp amplicon was digested with *Ase1* restriction enzyme (New England BioLabs, Massachusetts, USA) in a 30 µl digestion reaction consisting of 10 µl of the PCR product, 5 µl of NEB 3.1 10× Buffer (New England BioLabs, Massachusetts, USA), 10U *Ase1*, the reaction was made up to 30 µl with nuclease free water. Digestion proceeded at 37 °C for an hour. Wild type *SLCO1B1 c.1929A* yielded two fragments 282 and 188 bp (Fig. 1), the restriction site was abolished in the *SLCO1B1 c.1929C* variant yielded an undigested 470 bp fragment, whereas, the heterozygote *SLCO1B1 c.1929A*/*C* genotype yielded 3 fragments of 470, 282 and 188 bp. Digested fragments were identified using gel electrophoresis on a 3% Invitrogen UltraPure Agarose gel (ThermoFisher Scientific, Massachusetts, USA).

#### Validation of the RFLP method by Sanger sequencing

To validate the RFLP all sample amplicons were first cleaned in a 20 µl reaction that included 5 µl of the PCR product, 1U of FastAp thermosensitive alkaline phosphatase (ThermoScientific, UK), 2U Exonuclease 1 (ThermoScientific, UK), and incubated at 37 °C for 60 min followed by enzyme inactivation at 75 °C for 15 min using a MyCycler Thermal Cycler (Biorad, City, Country).

All cleaned amplicons were sequenced on an ABI 3100 Gene Analyser (Applied Biosystems, Massachusetts, USA) using a Big Dye Terminator Cycle Sequencing Ready Kit (Applied Biosystems, Massachusetts, USA). For each of the sequencing reaction, one of the primers used for PCR amplification was used or both were used individually to confirm variations.

#### Data analysis

Sequencing data was analysed using SeqMan Pro (DNASTAR Inc., Madison, USA). All statistical analysis was done using Graph Pad Prism (Graph Pad Software Inc, California, USA). Chi square was used to check for adherence to Hardy–Weinberg Equilibrium; differences in allele frequencies were analysed using the non-parametric One Way ANOVA (Kruskal Wallis test). Genotype associations with the phenotype peak serum drug concentration (Cmax), were analysed using the Student’s *t* test with Welch’s correction.

### Results and discussion

Allele and genotype frequencies are shown in Table 1. A total of 157 Zimbabwean samples were genotyped for the *SLCO1B1 c.1929A*>*C* SNP using both direct sequencing and the designed RFLP method. Comparison of results obtained using RFLP and direct sequencing showed a 100% correlation (see Fig. 1 showing an example of the comparison). All genotypes were consistent with Hardy–Weinberg equilibrium (P = 0.92). There were no statistically significant differences in the frequency of and *SLCO1B1 c.1929C* allele between the Zimbabwean population and all the major populations represented in the 1000 Genomes Project [7]. Amongst the 157 Zimbabwean samples genotyped, 30 individuals were healthy volunteers who participated in the rosuvastatin pharmacokinetic trial. Of these 30 healthy individuals, three individuals had the *SLCO1B1 c.1929C* allele, these 3 individuals also carried the *SLCO1B1 c.388G* allele and therefore bore the SLCO1B1*35 haplotype. The SLCO1B1*35 haplotype in this study population was 6%. None of the 157 individuals was homozygous for the *SLCO1B1 c.1929C* allele, the genotype *SLCO1B1 c.1929C/C* is rare even amongst all the ethnic populations represented in the 1000 Genomes Database (Table 1).

**Table 1:** Genotype distribution and relative frequencies of c.1929A>C in 30 healthy male Zimbabwean adults of Bantu ancestry

Population	Allele frequency		Genotype frequency		
	C	A	CC	AA	AC
This study	0.06	0.94	0.00	0.89	0.11
African	0.07	0.93	0.00	0.86	0.13
Caucasian	0.05	0.95	0.00	0.90	0.10
South Asian	0.05	0.95	0.00	0.90	0.10
East Asian	0.01	0.99	0.00	0.98	0.02

**Fig. 2:**
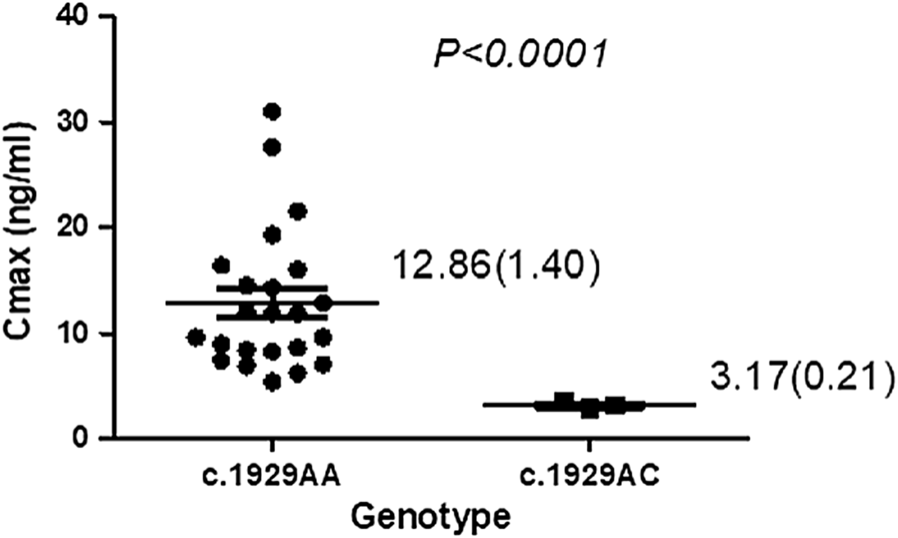
Association of the c.1929A>C variant with the pharmacokinetics of rosuvastatin in 30 healthy male Zimbabwean adults of Bantu ancestry. Graph shows Cmax mean per genotype with SM in bracket

The *SLCO1B1 c.1929A/C* genotype was associated with a 75% reduction in Cmax (P < 0.001) of rosuvastatin (Fig. 2) when compared to individuals with the *SLCO1B1 c.1929A/A* genotype. This reduction in Cmax in carriers of *SLCO1B1 c.1929C* allele could indicate a potential increase in hepatic uptake due to increased transporter activity. These results are consistent with the possible increased hepatic uptake by the OATP1B1 Phe^643^ variant reported previously for both methotrexate [2] and atorvastatin [3]. In addition, this study reports the possible importance of the *SLCO1B1 c.1929A*>*C* in interindividual response to rosuvastatin therapy. The liver serves as both the target of statin therapeutic activity and disposition therefore carriers of *SLCO1B1* c.1929.C variant are likely to have larger positive outcomes than non-carriers and possibly reduced incidence of SIM. However, questions remain on the possible impact of *SLCO1B1 c.1929A*>*C* on the pharmacotherapy of drugs like lopinavir and methotrexate, where increased hepatic uptake may potentially lower drug efficacy and therapeutic outputs.

### Conclusions

The RFLP method we describe here is quick, accurate and cost effective, it may assist in increasing evaluation of this important variant in a concerted effort to improve efficacious and safe use of drugs whose transport is mediated by the *SLCO1B1 transporter* OATP1B1.

## Limitations

The major limitation of this study was the sample size. However, the RFLP described here can be used to genotype larger sample sets as it is relatively cost effective when compared to current genotyping methods. In addition, the application of this RFLP in genotyping *SLCO1B1 c.1929A*>*C* in participants of the rosuvastatin pharmacokinetics trial launches the need for further investigation of this polymorphism and its effect on OATP1B1 transporter activity.

## Abbreviations

*SLCO1B1*: *Solute carrier organic anion transporting polypeptide IBI*
OATP1B1: organic anion transporting polypeptide 1B1
PCR: polymerase chain reaction
SNP: single nucleotide polymorphism
SIM: statin induced myopathy
Cmax: the maximum (or peak) serum concentration that a drug achieves in a specified compartment or test area of the body after the drug has been administrated
